# Active tyrosine phenol-lyase aggregates induced by terminally attached functional peptides in *Escherichia coli*

**DOI:** 10.1007/s10295-020-02294-4

**Published:** 2020-07-31

**Authors:** Hongmei Han, Weizhu Zeng, Guoqiang Zhang, Jingwen Zhou

**Affiliations:** 1 National Engineering Laboratory for Cereal Fermentation Technology, Jiangnan University, 1800 Lihu Road, Wuxi, 214122 Jiangsu China; 2 Key Laboratory of Industrial Biotechnology, Ministry of Education, School of Biotechnology, Jiangnan University, 1800 Lihu Road, Wuxi, 214122 Jiangsu China; 3 The Key Laboratory of Carbohydrate Chemistry and Biotechnology, Ministry of Education, Jiangnan University, 1800 Lihu Road, Wuxi, 214122 Jiangsu China; 4 Jiangsu Provisional Research Center for Bioactive Product Processing Technology, Jiangnan University, 1800 Lihu Road, Wuxi, 214122 Jiangsu China

**Keywords:** Self-assembling peptide, Tyrosine phenol-lyase, Active inclusion bodies, Thermostability, L-DOPA

## Abstract

The formation of inclusion bodies (IBs) without enzyme activity in bacterial research is generally undesirable. Researchers have attempted to recovery the enzyme activities of IBs, which are commonly known as active IBs. Tyrosine phenol-lyase (TPL) is an important enzyme that can convert pyruvate and phenol into 3,4-dihydroxyphenyl-l-alanine (L-DOPA) and IBs of TPL can commonly occur. To induce the correct folding and recover the enzyme activity of the IBs, peptides, such as ELK16, DKL6, L6KD, ELP10, ELP20, L6K2, EAK16, 18A, and GFIL16, were fused to the carboxyl terminus of TPL. The results showed that aggregate particles of TPL-DKL6, TPL-ELP10, TPL-EAK16, TPL-18A, and TPL-GFIL16 improved the enzyme activity by 40.9%, 50.7%, 48.9%, 86.6%, and 97.9%, respectively. The peptides TPL-DKL6, TPL-EAK16, TPL-18A, and TPL-GFIL16 displayed significantly improved thermostability compared with TPL. L-DOPA titer of TPL-ELP10, TPL-EAK16, TPL-18A, and TPL-GFIL16, with cells reaching 37.8 g/L, 53.8 g/L, 37.5 g/L, and 29.1 g/L, had an improvement of 111%, 201%, 109%, and 63%, respectively. A higher activity and L-DOPA titer of the TPL-EAK16 could be valuable for its industrial application to biosynthesize L-DOPA.

**Electronic supplementary material:**

The online version of this article (10.1007/s10295-020-02294-4) contains supplementary material, which is available to authorized users.

## Introduction

Tyrosine phenol-lyase (TPL) (EC4.1.99.2), a tetrameric enzyme, can catalyze stereospecific isotope exchange of *α*-protons of various amino acids with pyridoxal-5′-phosphate (PLP) as the cofactor [20]. TPL has been mainly isolated and characterized from bacteria, such as *Citrobacter freundii* [38,46], *Erwinia herbicola* [58], and *Fusobacterium nucleatum* [62]. Monovalent cations, K^+^ or NH_4_^+^ are necessary for achieving high activity levels of TPL [33]. The biosynthesis based on the enzyme activity of TPL has attracted attention for its application in the production of enantiomerically pure *α*-deuterated (*S*)-amino acids, such as S-alkyl/aryl-cysteines [7] and l-dihydroxyphenylalanine (L-DOPA) [54]. L-DOPA, as a precursor of dopamine, has been regarded as the main medicine to treat Parkinson’s disease since the 1960s [42].TPL can catalyze the reversible hydrolytic cleavage of L-tyrosine to phenol and ammonium pyruvate [8], and due to the reversibility of this reaction, when the catechol is substituted for phenol, L-DOPA is synthesized. The presence of ammonium salt plays an important role in leading the direction of the reversible catalysis in the production of L-DOPA [57].

Metabolic engineering strategies for de novo synthesis of L-DOPA have mainly focused on directing major carbon fluxes toward desired L-tyrosine formation [50]. L-tyrosine, as a precursor for synthesis of L-DOPA, is usually difficult to be synthesized and accumulated by metabolic pathways [10]. There are inevitable issues for de novo synthesis of L-DOPA, such as low titer and browning caused by oxidation and remaining tyrosine; therefore, research is important to further improve the catalytic performance of TPL and the enzymatic synthesis of L-DOPA. In the last decade, production of pyruvic acid, which is the precursor for enzymatic synthesis of L-DOPA, has been significantly improved [64] [29–31]. This has significantly lowered the price of pyruvic acid and makes the enzyme synthesis of L-DOPA the most cost-effective method. Higher enzyme activity and operational stability are research hotspots in the development of the industrial application of TPL [39]. Thermostability and enzyme activity of the TPL from *Symbiobacterium toebii* were simultaneously improved by the application of random mutagenesis and the subsequent reassembly of the acquired mutations [43]. It was found that Ser51 of TPL participated in stabilizing the ammonium form of Lys257, by studying the kinetics for the complexes of the mutant form with competitive inhibitors [1]. It was also found that Phe-448 and Phe-449 in TPL contributed to β-elimination catalysis reactions by introducing ground state destabilization in the substrate [39].

Heterogeneous proteins expressed in recombinant bacteria aggregate as insoluble protein clusters were defined as inclusion bodies (IBs) [26]. These IBs are formed due to unfolded or highly misfolded polypeptides that fail to reach their normal conformation [48]. Refolding of proteins from IBs is affected by several factors, including solubilization of IBs by denaturants, removal of the denaturant, and induction of refolding by small molecule additives [13,26]. Some protein aggregates, known as active IBs, become functionally active proteins due to a correct fold with the assistance of self-assembly peptides (SAPs) [53,61] and there are also studies showing that these SAPs could enhance the thermostability of the target enzyme [22]. SAPs, as specific type of peptide, can spontaneously assemble into nanostructures. Moreover, SAPs displayed several advantages, such as small size, convenient operations, and cost-saving. In last several years, these SAPs have been applied to recover the biological activity of bacterial IBs. Active IBs induced by terminally attaching SAPs have also been successfully applied in protein purification with high yields and purity [9,49,60].

In this study, TPL was overexpressed in *Escherichia coli* BL21(DE3) as a heterogeneous protein. Besides the soluble expression of TPL, insoluble TPL aggregates without enzyme activity also existed, which might be resulted from undesirable misfolded polypeptides. As the waste byproducts during protein expression, IBs are recognized as the major bottleneck in recombinant protein expression [19] and are discarded from further processing or are eventually used as a pure protein by in vitro refolding and recovery [37]. As whole-cell biocatalyst, L-DOPA titer could be seriously affected by TPL IBs without activity. Therefore, it is necessary to recover active proteins from the TPL IBs and improve the catalytic performance by fusing SAPs to the C-terminus of TPL. The short peptides ELK16, DKL6, L6KD, ELP10, ELP20, L6K2, EAK16, 18A, and GFIL16 were each attached to the C-terminus of TPL, respectively. Amino acid sequences of peptides were showed in Table S1. It was found that TPL-ELP10, TPL-EAK16, TPL-18A, and TPL-GFIL16 showed obvious advantages, such as IBs with enzyme activity and improved thermostability and the L-DOPA titer of TPL-EAK16 increased to 53.8 g/L. As a result, fusion of peptides to the C-terminus of TPL has been identified as achieving improved enzyme performance. The enzyme activity of TPL IBs was first studied and activated using peptides, demonstrating the potential of peptides as novel IB-inducing fusion tags in vivo. In addition, peptides could also be successfully applied in the production and purification of proteins.

## Materials and methods

### Construction of the plasmids

Strains of *E. coli* BL21(DE3) and *E. coli* JM109, pET28a-TPL plasmid were preserved in the laboratory [14]. The nucleotide sequence of TPL gene has been recorded in National Center for Biotechnology Information (NCBI) and GenBank accession number was MN205565. Proteins expressed by plasmid pET28a-TPL in *E. coli* BL21 were designated as TPL. Plasmid pET28a-TPL was amplified using the polymerase chain reaction (PCR) with pET-TPL-F/R primers (Table S2). Genes of peptides were synthesized by GenScript (Nanjing, China). The synthesized genes were amplified by PCR with primers (Table S2) and assembled into pET28a-TPL with homologous recombination to yield vector pET28a-TPL-ELK16, pET28a-TPL-DKL6, pET28a-TPL-L6KD, pET28a-TPL-ELP10, pET28a-TPL-ELP20, pET28a-TPL-L6K2, pET28a-TPL-EAK16, pET28a-TPL-18A, and pET28a-TPL-GFIL16. The corresponding proteins expressed by these plasmids were designated as TPL-ELK16, TPL-DKL6, TPL-L6KD, TPL-ELP10, TPL-ELP20, TPL-L6K2, TPL-EAK16, TPL-18A, and TPL-GFIL16, respectively.

### Expression of TPL

The growth of recombinant cells was carried out in terrific broth (TB) medium supplemented with 50 mg/L kanamycin with 400 rpm at 37 °C in 3 L bioreactor (T&J Bio-engineering Co., LTD, Shanghai, China). Isopropyl-*β*-d-thiogalactoside (IPTG) with a final concentration of 0.2 mM was added to the culture to induce the expression of proteins and the cells were cultured for a further 10 h at 20 °C.

### Preparation and SDS-PAGE analysis of TPL

The collected cell pellets were washed three times and resuspended with buffer B1 [50 mM KH_2_PO_4_–K_2_HPO_4_, 1% (v/v) glycerol, (pH 8.5)]. Cell suspensions were lysed by ultrasonic disruption. The resulting cell lysates were centrifuged at 10,000 rpm for 10 min at 4 °C to separate the soluble fractions and insoluble fractions. The amounts of fusion proteins in both soluble and insoluble fractions were analyzed by sodium dodecyl sulfate-polyacrylamide gel electrophoresis (SDS-PAGE). Protein concentration was normalized using an enhanced BCA protein assay kit (P0009; Beyotime Biotechnology, Jiangsu, China).

The resulting TPLs all containing a 6 × His fusion tag, were purified by an His-Trap FF 5-mL column with AKTA Pure system (GE Healthcare, Piscataway, NJ, USA). The purification operation was the same as a previous study [14].

### Enzyme activity assay of TPL

The relative enzyme activity of supernatant and precipitates of TPLs was determined with the *β*-elimination reactions of L-tyrosine [2]. L-tyrosine was decomposed into phenol, pyruvate, and ammonia, and the resulting pyruvate concentration was measured by Agilent 1200–series high-performance liquid chromatography (HPLC) [28]. To determine the optimal pH value of TPLs at 20 °C, L-tyrosine was dissolved in 50 mM KH_2_PO_4_-K_2_HPO_4_ (pH 6 to 8) and 50 mM glycine–NaOH (pH 8–10) buffer, respectively. The enzyme activity of intracellular supernatants from TPL were defined as 100%. The enzyme activity of TPLs was determined by the same method at different temperatures, as one unit of TPL activity, defined as the amount of enzyme producing 1 µM pyruvate per min. The half-life of TPLs at 20 °C, 40 °C was determined as before [14]. A reference standard of 100% TPLs without maintenance at 20 °C, 40 °C, or 60 °C was established and measured at the corresponding reaction temperature.

The kinetic parameters of TPLs were determined at 20 °C, with 900 μL substrate and 100 μL enzyme liquid-containing 30 µM PLP. The substrate was L-DOPA with a gradient concentration from 0.1 to 6 mM. Lineweaver–Burk plots were generated for the analysis of *K*_*m*_, *V*_max_, and *k*_cat_*/K*_*m*_ [3].

### Whole-cell biosynthesis

The initial reaction mixture was composed of 18 g/L sodium pyruvate, 10 g/L catechol, 30 g/L ammonium salt, 4 g/L sodium sulfite, 2 g/L EDTA, and 30 µM PLP. The pH value of reaction mixture was adjusted to 8.5 with ammonium hydroxide. Sodium pyruvate was added at 12 g/L/h from 0 to 5 h. Catechol was added at 10 g/L/h during the first 2 h, at 8 g/L/h from 2 to 4 h, and at 4 g/L/h from 4 to 5 h. The reaction was at 15 °C. Ammonium salt can trigger the reaction to the direction of synthesizing L-DOPA due to the reversibility of reaction [34]. It was necessary to avoid light during the reaction to protect the production L-DOPA from being decomposed. Sample was extracted every 1 h and treated with the 50% (v/v) 2 M HCL.

### HPLC analysis

L-DOPA was analyzed by an Agilent 1200-series HPLC system equipped with a reverse-phase Gemini NX-C18 column (4.6 × 250 mm) according to a previous protocol [15].

### Transmission electron microscopic analysis

Morphometric analysis of the TPLs was performed using transmission electron microscopy (TEM). Solutions of 2.5% glutaraldehyde and 2% osmium tetroxide were used successively to fix the cells. After a graded ethanol serial dehydration step, the cells were embedded in epoxy resins. The fixed cells were sectioned, stained by uranyl acetate solution and lead citrate, and were observed using a Hitachi H-7650B (Hitachi, Japan) TEM at an accelerating voltage of 80 kV.

## Results

### Formation of active TPL IBs in *E. coli*

**Fig. 1:**
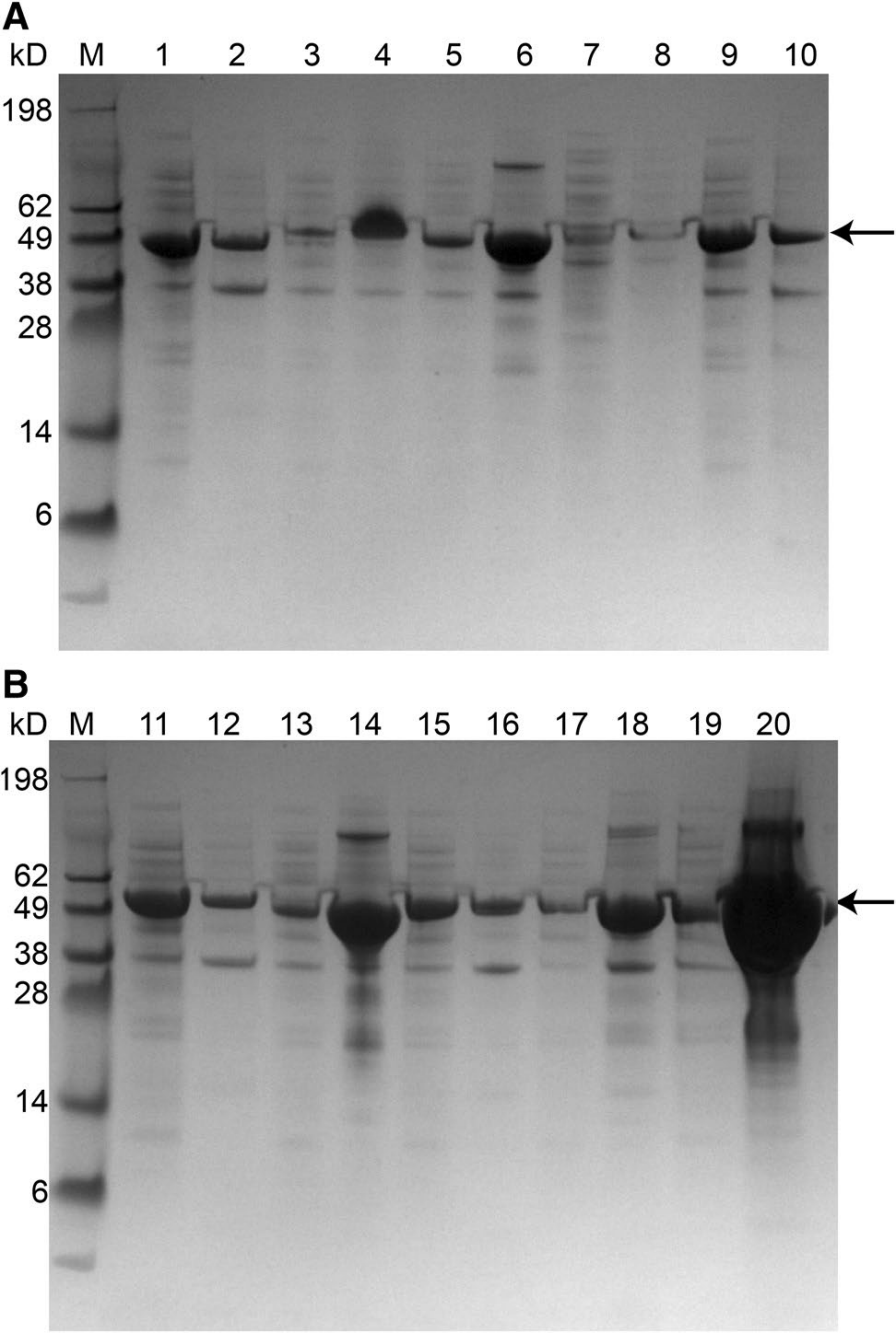
SDS-PAGE analysis of fusion protein expression. Intracellular supernatant of TPL (lane 1); aggregate of TPL (lane 2), TPL-ELK16 (lane 4), TPL-DKL6 (lane 6), TPL-L6KD (lane 8), TPL-ELP10 (lane 10), TPL-ELP20 (lane 12), TPL-L6K2 (lane 14), TPL-EAK16 (lane 16) and TPL-18A (lane 18), TPL-GFIL16 (lane 20); supernatant of TPL-ELK16 (lane 3), TPL-DKL6 (lane 5), TPL-L6KD (lane 7), TPL-ELP10 (lane 9), TPL-ELP20 (lane 11), TPL-L6K2 (lane 13), TPL-EAK16 (lane 15), TPL-18A (lane 17), and TPL-GFIL16 (lane 19). Levels were normalized according to a 4-mg standard. Targets are denoted with a black arrow. M, standards (kDa)

**Fig. 2:**
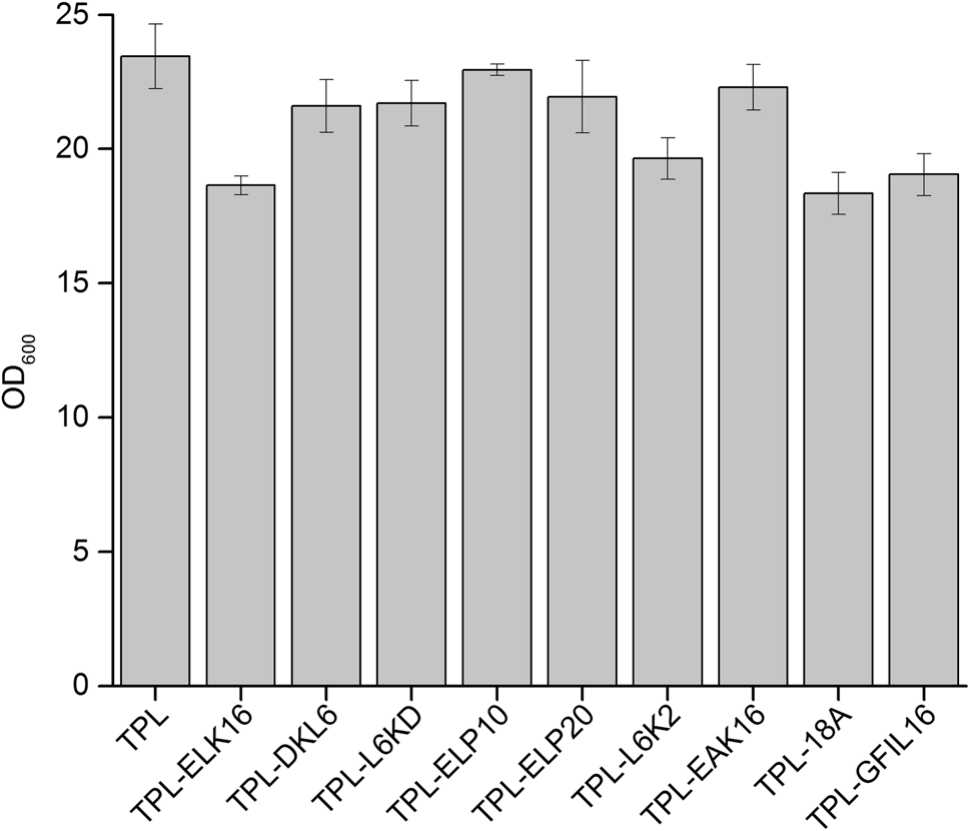
The final OD_600_ of *E. coli* BL21 expressing TPLs. *E. coli* BL21(DE3) cells expressing TPL, TPL-ELK16, TPL-DKL6, TPL-L6KD, TPL-ELP10, TPL-ELP20, TPL-L6K2, TPL-EAK16, TPL-18A, and TPL-GFIL16 were cultivated at 37 °C for 2 h. The temperature was adjusted to 20 °C for 10 h. Experiments were performed in triplicate, and error bars represent the standard deviation

The TPL without a short peptide displayed two expression styles as supernatant and aggregate (52 kDa) (Fig. 1). SDS-PAGE results showed that TPL-ELK16, TPL-DKL6, TPL-L6K2, TPL-18A, and TPL-GFIL16 were mainly expressed in the insoluble fractions. The growth of *E. coli* BL21 containing fusion proteins was slow compared to the *E. coli* BL21 containing TPL (Fig. 2). The final OD_600_ of *E. coli* BL21 expressing TPL-ELK16, TPL-L6K2, TPL-18A, and TPL-GFIL16 was 18.7, 19.6, 18.4, and 19.2, which was 4.8, 3.9, 5.1, and 4.4 less, respectively, for the *E. coli* BL21 expressing TPL. To a certain extent, TPLs that were fused with peptide and expressed in the form of aggregate particles had a slight inhibitory effect on the *E. coli* BL21 cell growth.

### Catalytic properties of TPLs

**Fig. 3:**
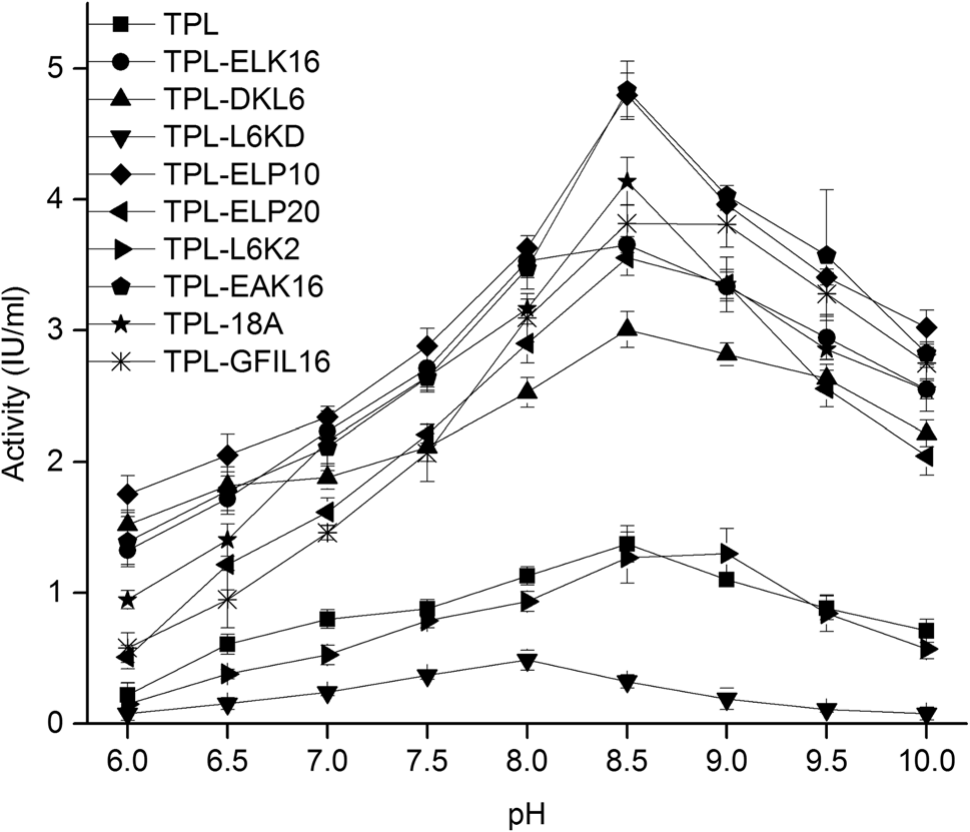
Effects of pH on TPL and TPL fused with peptides on activity. Enzyme activity at pH from 6.0 to 10. The temperature of the reaction was at 20 °C. Experiments were performed in triplicate. Error bars represent the standard deviation

**Fig. 4:**
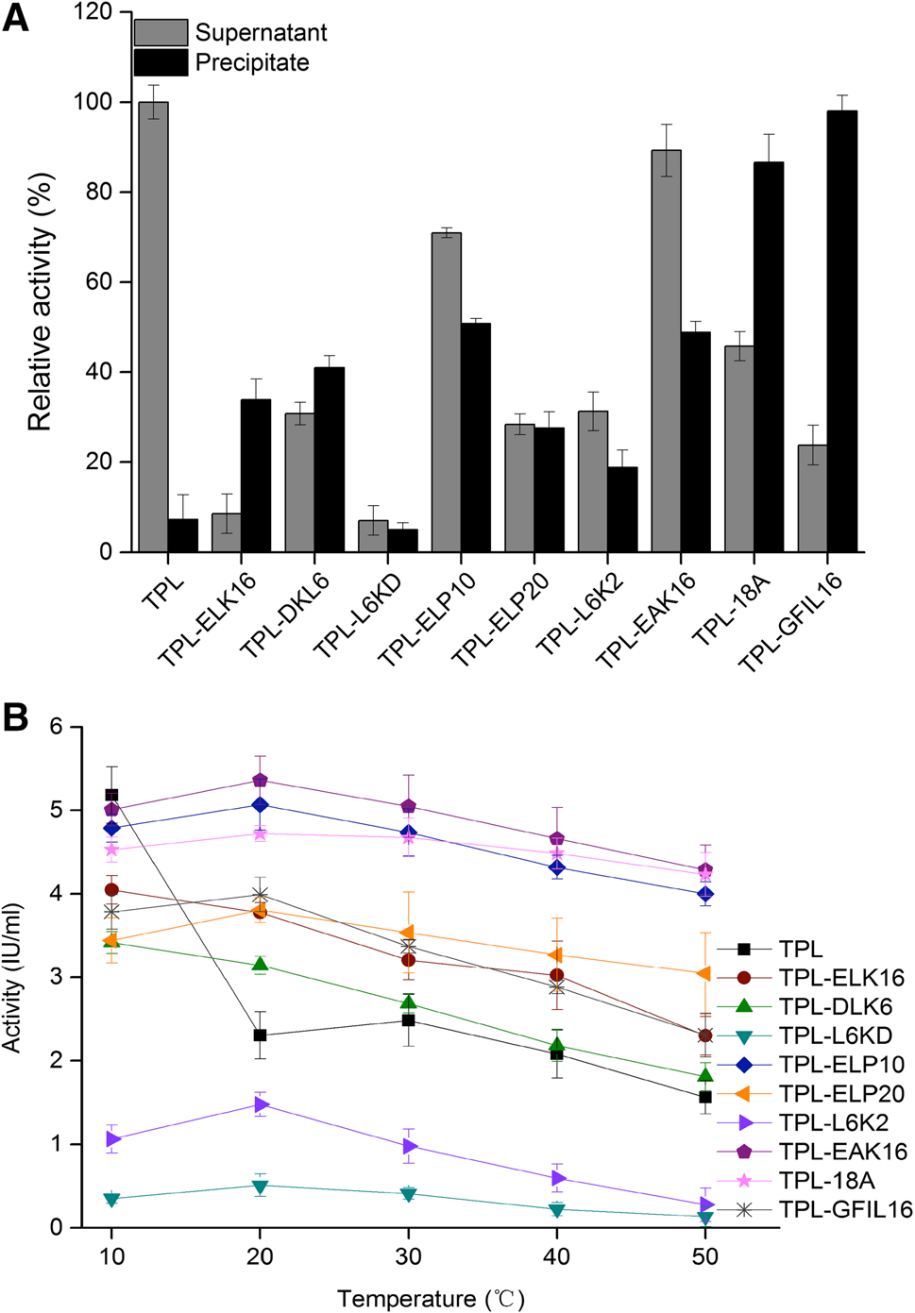
Enzyme activity of TPL and TPL fused with peptides. Relative activity of supernatants and precipitates from TPL and TPL fused with peptides at 20 °C (**a**). Enzyme activity of TPL and TPL fused with peptides at 10 °C, 20 °C, 30 °C, 40 °C, and 50 °C (**b**). Experiments were performed in triplicate, and error bars represent the standard deviation

The optimal pH value for the catalysis of TPL-ELK16, TPL-DKL6, TPL-L6KD, TPL-ELP10, TPL-ELP20, TPL-EAK16, and TPL-18A was 8.5. For the catalysis of TPL-L6K2 and TPL-GFIL16, the optimal pH value was 9.0 (Fig. 3). Anyway, alkaline buffer is a good fit for TPLs. Aggregate particles of TPL displayed only 7.3% enzyme activity in comparison with TPL supernatant (Fig. 4a); compared with aggregate particles of TPL-DKL6, TPL-ELP10, TPL-EAK16, TPL-18A, and TPL-GFIL16, this displayed 40.9%, 50.7%, 48.9%, 86.6%, and 97.9% enzyme activity, respectively. TPL displayed maximal enzyme activity of 5.1 IU/mL at 10 °C, (Fig. 4b), but this decreased by 55.8% at 20 °C. The obvious decrease of enzyme activity has been verified in other study [14], which could be contributed to the lower thermostability. TPL does not belong to enzyme with good thermostability. Therefore, reaction catalyzed by TPL was generally performed at 15 °C [62]. The maximal enzyme activity of TPL-ELP10, TPL-EAK16, TPL-18A, and TPL-GFIL16 was 5.0 IU/mL, 5.4 IU/mL, 4.7 IU/mL, and 3.9 IU/mL, respectively, exhibiting improvement by 117.4%, 133.0%, 104.3%, and 69.5% relative to TPL at 20 °C. Using L-DOPA as the substrate, kinetic parameters showed that the *V*_max_ and *K*_*m*_ values of TPL were 0.04 mM/min/mg and 2.19 mM (Table S3). The *V*_max_ of TPL-DKL6, TPL-ELP10, TPL-EAK16, TPL-18A, and TPL-GFIL16 was higher by 24.4%, 24.4%, 12.2%, 9.8%, and 17%, respectively, relative to the TPL. The *K*_*m*_ value of TPL-DKL6, TPL-ELP10, TPL-EAK16, and TPL-GFIL16 increased by 5.5%, 22.3%, 7.8%, and 1.8%, respectively, relative to the TPL. The *k*_cat_*/K*_*m*_ value of TPL-DKL6, TPL-ELP10, TPL-EAK16, and TPL-GFIL16 increased by 25.4%, 24.0%, 11.1%, and 17.7%, respectively, relative to the TPL.

### Thermostability of TPLs at different temperatures

**Fig. 5:**
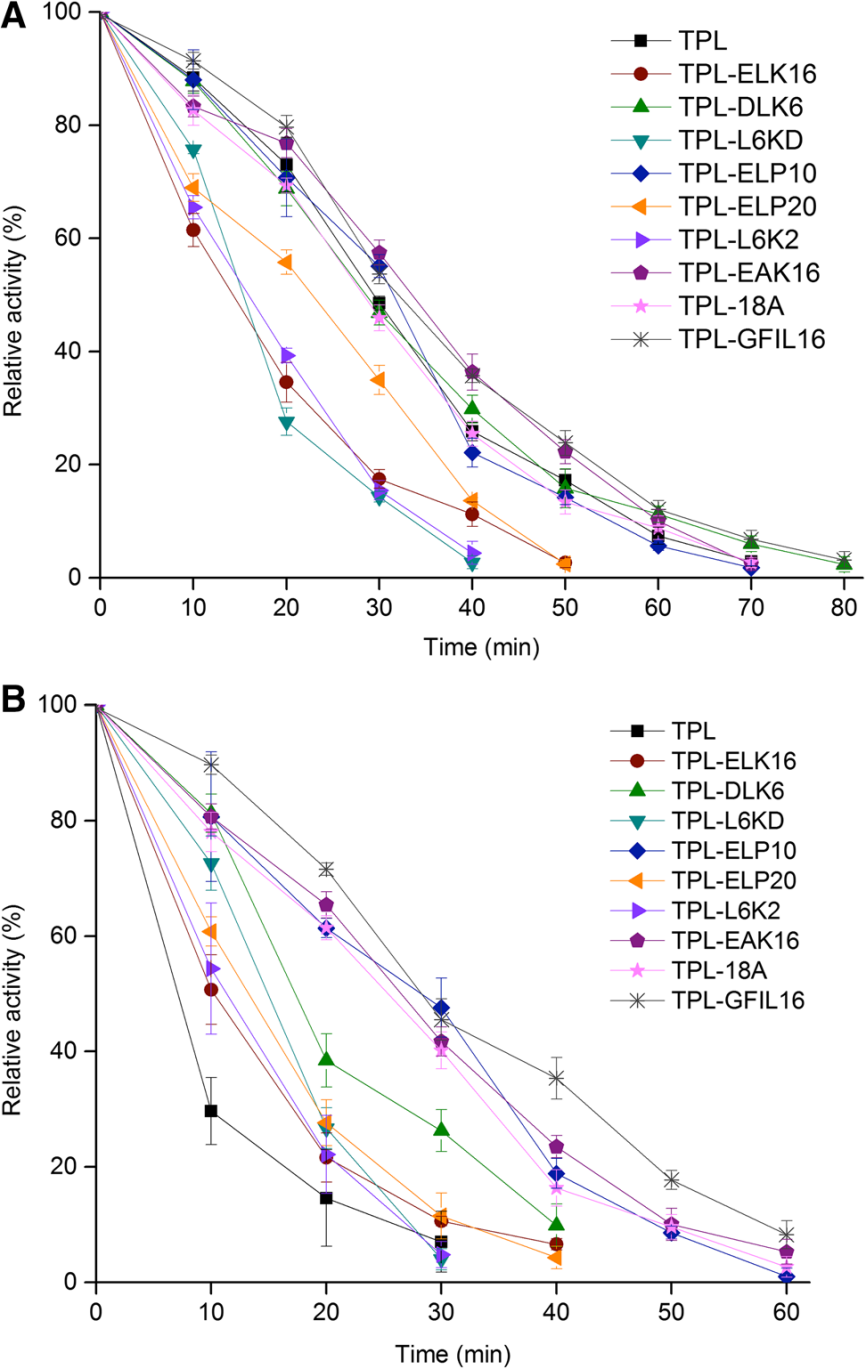
Relative activity of TPL and TPL fused with peptides. Relative activity of TPL, TPL-ELK16, TPL-DKL6, TPL-L6KD, TPL-ELP10, TPL-ELP20, TPL-L6K2, TPL-EAK16, TPL-18A, and TPL-GFIL16 at 20 °C (**a**) and 40 °C (**b**). Experiments were performed in triplicate, and error bars represent the standard deviation

The tests showed that TPL-DKL6, TPL-EAK16, TPL-18A, and TPL-GFIL16 displayed thermostability with a half-life of 14.8 min, 14.2 min, 14.1 min, and 15.8 min at 20 °C and 12.0 min, 13.9 min, 11.6 min, and 13.9 min at 40 °C, respectively. The residual activity for TPL remained at only 29.6% following incubation at 40 °C for 10 min (Fig. 5). The half-life of TPL was 13.8 min at 20 °C and 6.3 min at 40 °C. All the results demonstrated that the fusion of functional short peptides increased the thermostability of TPL significantly, especially TPL-18A and TPL-GFIL16.

### L-DOPA titer by whole-cell biosynthesis

**Fig. 6:**
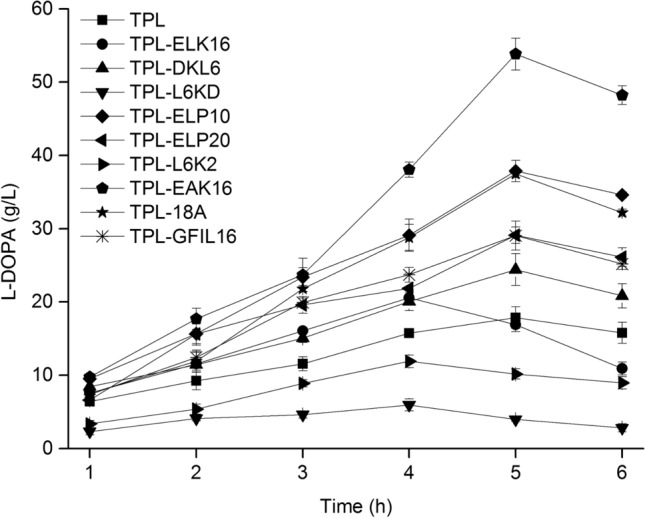
Effect of peptides on L-DOPA production by whole-cell biosynthesis. Whole-cell L-DOPA production of TPL, TPL-ELK16, TPL-DKL6, TPL-L6KD, TPL-ELP10, TPL-ELP20, TPL-L6K2, TPL-EAK16, TPL-18A, and TPL-GFIL16 at 20 °C. Experiments were performed in duplicate, and error bars represent the standard deviation

Whole-cell biosynthesis was carried out for 6 h at 20 °C and the L-DOPA titer was tested every hour (Fig. 6). As the reaction progressed, crystals of L-DOPA were observed. When the reaction time reached 5 h at 20 °C, L-DOPA biosynthesized by TPL cells was 17.9 g/L. In contrast, L-DOPA biosynthesized by TPL-ELP10, TPL-EAK16, TPL-18A, and TPL-GFIL16 cells reached maximal values of 37.8 g/L, 53.8 g/L, 37.5 g/L, and 29.1 g/L, respectively, showing improvement by 111%, 201%, 109%, 63%, respectively, relative to TPL. The improved L-DOPA titer was correlated with improved enzyme activity. Moreover, the expression of soluble TPL-ELP10 and TPL-EAK16 has been enhanced, which could be observed on the SDS-PAGE (Fig. 1). IBs of TPL-ELP10, TPL-EAK16, TPL-18A, and TPL-GFIL16 also displayed catalytic activity (Fig. 4a). Both the increased expression of soluble TPL and active inclusion bodies probably resulted in the increased L-DOPA titer.

### Morphology analysis of TPLs

**Fig. 7:**
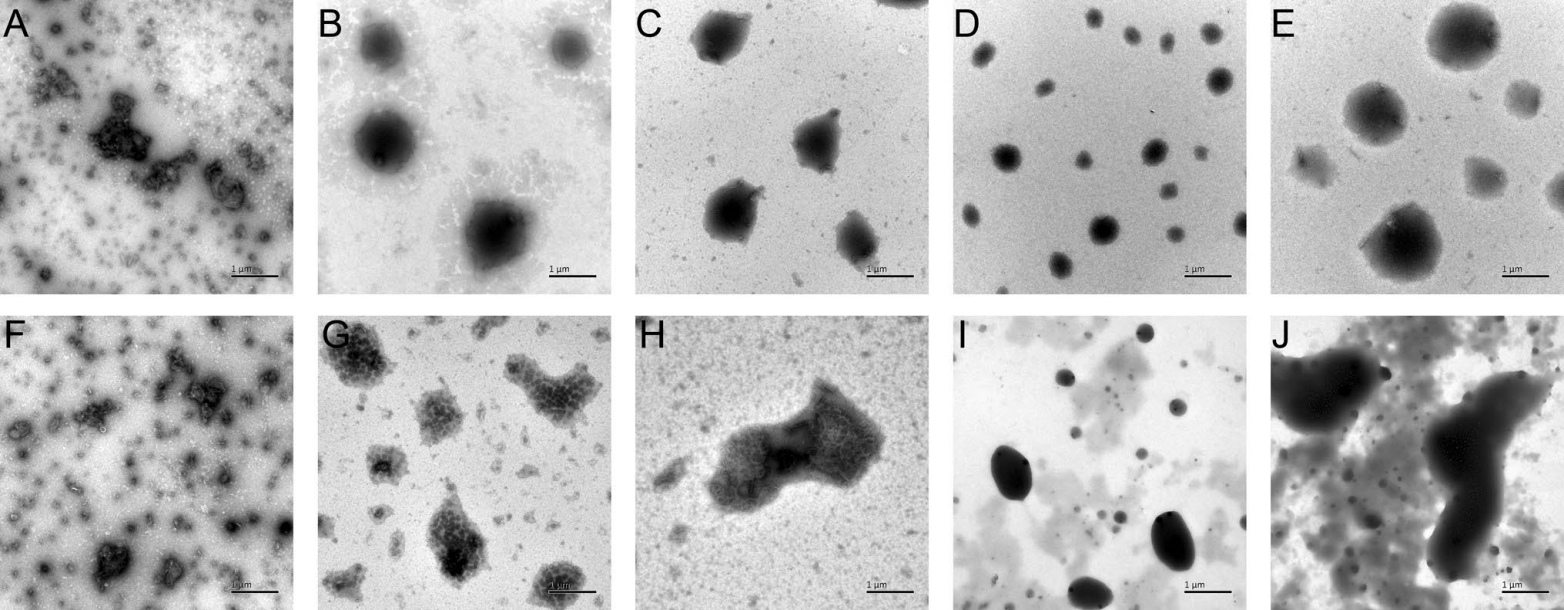
Negative stained transmission electron micrographs (TEM) images of TPLs. TEM of TPL (**a**), TPL-ELK16 (**b**), TPL-DKL6 (**c**), TPL-L6KD (**d**), TPL-ELP10 (**e**), TPL-ELP20 (**f**), TPL-L6K2 (**g**), TPL-EAK16 (**h**), TPL-18A (**i**), and TPL-GFIL16 (**j**). The magnification was 15,000 × . Rule bar represents 1 µm

Negative stained TEM images showed homo-oligomeric structures for the freshly prepared TPL, TPL-ELK16, TPL-DKL6, TPL-L6KD, TPL-ELP10, TPL-ELP20, TPL-L6K2, TPL-EAK16, TPL-18A, and TPL-GFIL16 from *E. coli* BL21 (Fig. 7). The surfactant-like peptides DKL6, L6KD, and L6K2 can act as a pull-down handler for converting soluble proteins into active aggregates and ELP covalently attaches with the C-terminus residue of the xylanase via a random coil [[22]]. The self-assembly of EAK16-family peptides can induce fibrillary or globular assemblies [9]. ELK16, DKL6, L6KD, with ELP10 induce the formation of spherical TPL aggregates and L6K2, EAK16 induce irregular small TPL aggregates. It has been shown that 18A can self-assemble by coiled formation in the aqueous solution, and form discoidal particles, or helix fibrils [21]. Accordingly, elliptic aggregates of TPL-18A were formed. GFIL16 is comprised of hydrophobic residues and has strong hydrophobicity [49]. Therefore, results showed that GFIL16 induced the formation of large and amorphous TPL aggregates that were different from the others.

## Discussion

There are a few routine methods reported to improve the catalytic efficiency of TPL-mediated biosynthesis. It is common to genetically engineer the existing enzymes by rational protein design or random/saturated mutagenesis [41], which are labor-intensive and time-consuming. To improve thermostability and reusability, immobilization technology is a useful tool [35]. Conventional immobilization usually leads to the conformational changes or to prevention of the active pocket from being bound with substrates, and this could result in a decrease in enzyme activity [24]. TPL immobilized with wet nanoporous silica gels resulted in modified steady-state distribution of catalytic intermediates, but specific activities decreased [44]. However, SAPs were covalently attached with the C-terminus residue of the TPL via an irregular coil, overcoming the conflict between stability and enzyme activity. For example, 18A fused nitrilase aggregates (Nit-SEA) displayed enzyme activity and increased thermostability [55], which was the prospective results in this study. SAPs were able to serve as “pull-down” fusion tags to effectively induce the formation of cytoplasmic active IBs in *E. coli*, such as 18A, ELK16, DKL6, L6KD, and L6K2 [11]. EAK16 and GFIL16, which were composed of hydrophobic amino acids, played functional roles in the formation of active IBs due to strong hydrophobicity [1]. Accordingly, aggregate particles of TPL-DKL6, TPL-ELP10, TPL-EAK16, TPL-18A, and TPL-GFIL16 displayed 40.9%, 50.7%, 48.9%, 86.6%, and 97.9% enzyme activity relative to TPL supernatant, respectively. Moreover, TPL-DKL6, TPL-EAK16, TPL-18A, and TPL-GFIL16 displayed thermostability with prolonged half-life.

Previously investigated SAPs include ionic beta-strand peptide ELK16 [51], elastin-like polypeptide (ELP) [47], surfactant-like peptide (DKL6, L6KD, and L6K2) [63], charged and hydrophobic peptide EAK16 [9], modified apolipoprotein A-I mimetic amphipathic peptide 18A [25], and hydrophobic self-assembling peptide GFIL16 [49]. The SAPs that induced the formation of active IBs were regarded as the carrier for immobilization. IBs with high specific enzyme activity have been verified as potentially useful biocatalysts [36]. Target proteins were successfully released from active IBs upon cleavage of the intein between the peptide tag and the target protein [27]. It has become a promising method to develop a quick technique to enable protein expression and purification in bacteria. Pure enzyme and immobilized enzymes have been mainly used as biocatalyst in current industrial production. Compared to the traditional purification and immobilization, this process could achieve both time-saving and cost-saving in industrial application [52]. It has also been proven that one-step purification is feasible by inserting an appropriate self-cleavable site between the target protein and the peptide [17].

*E. coli* has served as a cell factory for recombinant protein expression for a long time and the cells expressing an enzyme can be used as biocatalysts [4,5], but overexpression of heterogeneous proteins can lead to the formation and accumulation of inactive IBs due to misfolded polypeptides [12,59]. Currently effective strategies have been developed to recover enzyme activity from aggregate particles [23,56]. Active IBs have shown unique advantages, such as easy separation, greater stability, and reusability, and offer robustness in applications [32]. Fusion of the N-terminal peptide GFIL8 to the Ulp1 results in increased production of active IBs with higher resistance to limited proteolysis and less leakage at different storage temperatures [18]. In this study, peptide-induced aggregates did not interfere with the correct folding of the target proteins, and active TPL IBs also contributed to the improved enzyme activity and thermostability.

In summary, more attention should be paid to recovering the enzyme activity of IBs with the assistance of SAPs [6,61]. To improve the biosynthesis of L-DOPA, TPL-EAK16 with obvious advantages was achieved with the help of EAK16 SAPs. The L-DOPA titer and productivity of TPL-EAK16 were 53.8 g/L and 10.76 g/L/h, respectively. Compared with the previous reported aggregation prone fusion partners [40,45], the peptides used in this study were much shorter in size and more easily modulated due to their simpler secondary structure [16]. However, it is difficult to control the amount and size of aggregate particles in the host cell. Spherical TPL aggregates were induced by ELK16, DKL6, L6KD, and ELP10. Aggregates of TPL-L6K2, TPL-EAK16 displayed small irregular shape. GFIL16 with strong hydrophobicity induced the formation of large and amorphous TPL aggregates. All the results demonstrated that TPL aggregate particles with enzyme activity induced by the SAPs had significance for application to other enzymes. Moreover, IBs, as biocatalysts, were easily separated and purified [11]. This method is, therefore, much easier, more economic, and time-efficient to scale up from an economic point of view.

## Electronic supplementary material

Below is the link to the electronic supplementary material.

Supplementary file1 (PDF 90 kb)
