# Supplementary material for: Active tyrosine phenol-lyase aggregates induced by terminally attached functional peptides in Escherichia coli
Source: J Ind Microbiol Biotechnol. 2020 Jul 31;47(8):563–71. doi: 10.1007/s10295-020-02294-4 (PMC7508748; doi:10.1007/s10295-020-02294-4)
Supplement: Supplementary file 1 — Supplementary file1 (PDF 90 kb) [file 10295_2020_2294_MOESM1_ESM.pdf]

Supplementary material Table S1, Table S2, Table S3

Journal: **Journal of Industrial Microbiology and Biotechnology**

Title: **Active Tyrosine Phenol-lyase Aggregates induced by terminally attached Functional Peptides in *Escherichia coli***

Author: Hongmei Han [1,2], Weizhu Zeng[2,4], Guoqiang Zhang[1,2], Jingwen Zhou[1,2,4*]

Author affiliation:

[1] National Engineering Laboratory for Cereal Fermentation Technology, Jiangnan University, 1800 Lihu Road, Wuxi, Jiangsu 214122, China;

[2] Key Laboratory of Industrial Biotechnology, Ministry of Education, School of Biotechnology, Jiangnan University, 1800 Lihu Road, Wuxi, Jiangsu 214122, China;

[3] The Key Laboratory of Carbohydrate Chemistry and Biotechnology, Ministry of Education, Jiangnan University, 1800 Lihu Road, Wuxi, Jiangsu 214122, China;

[4] Jiangsu Provisional Research Center for Bioactive Product Processing Technology, Jiangnan University, 1800 Lihu Road, Wuxi, Jiangsu 214122, China.

***Corresponding author:**

Jingwen Zhou

Mailing address: School of Biotechnology, Jiangnan University, 1800 Lihu Road, Wuxi, Jiangsu 214122, China

Phone: +86-510-85914371, Fax: +86-510-85914371

E-mail: zhoujw1982@jiangnan.edu.cn.

Tables

Table S1 Amino acid sequences of peptides

Peptides	Amino acids	Products
ELK16	LELELKLKLELELKLK	TPL-ELK16
DKL6	DKLLLLLL	TPL-DKL6
L6KD	LLLLLLKD	TPL-L6KD
ELP10	VPGVGVPGVG	TPL-ELP10
ELP20	VPGVGVPGVGVPGVGVPGVG	TPL-ELP20
L6K2	LLLLLLKK	TPL-L6K2
EAK16	AEAEAKAKAEAEAKAK	TPL-EAK16
18A	EWLKAFYEKVLEKLKELF	TPL-18A
GFIL16	GFILGFILGFILGFIL	TPL-GFIL16

Table S2 Oligonucleotides used in this study

Primer	Sequence (5'-3')
pET-TPL-F	TAAAAGCTTGCGGCC
pET-TPL-R	GATATAGTCGAAGCG
TPL-ELK16-F	CGCTTCGACTATATCCCGACCCCGCCGAC
TPL-ELK16-R	GGCCGCAAGCTTTTATTTCAGTTTAAGC
TPL-DKL6-F	CGCTTCGACTATATCCCGACCCCGCCGAC
TPL-DKL6-R	GGCCGCAAGCTTTTACAGTAACAACAA
TPL-L6KD-F	CGCTTCGACTATATCCCGACCCCGCCGAC
TPL-L6KD-R	GGCCGCAAGCTTTTAATCTTTCAGTAAC
TPL-ELP10-F	CGCTTCGACTATATCCCGACCCCGCCGAC
TPL-ELP10-R	GGCCGCAAGCTTTTAACCAACCCCAGG
TPL-ELP20-F	CGCTTCGACTATATCCCGACCCCGCCGAC
TPL-ELP20-R	GGCCGCAAGCTTTTATCCAACGCCAGGAAC
TPL-L6K2-F	CGCTTCGACTATATCCCGACCCCGCCGAC
TPL-L6K2-R	GGCCGCAAGCTTTTATTTTTTCAGAAGC
TPL-EAK16-F	CGCTTCGACTATATCCCGACCCCGCCGAC
TPL-EAK16-R	GGCCGCAAGCTTTTACTTGGCTTTTGCTTCAG
TPL-GFIL16-F	CGCTTCGACTATATCCCGACCCCGCCGAC
TPL-GFIL16-R	GGCCGCAAGCTTTTACAGGATGAAACCC
TPL-18A-F	CGCTTCGACTATATCCCGACCCCGCCGAC
TPL-18A –R	GGCCGCAAGCTTTTA AAACAGTTCCTTC

*Underlined bases represent homologous arm.

Table S3 The kinetic characteristics of TPLs with L-DOPA substrate

TPL*	V_{max} (mM × min^{-1})	K_m (mM)	K_{cat} (s^{-1})
TPL	0.041±0.0042	2.19±0.119	0.35±0.036
TPL-ELK16	0.020±0.0015	4.18±1.607	0.174±0.013
TPL-DKL6	0.051±0.0024	2.32±0.504	0.439±0.020
TPL-L6KD	-	-	-
TPL-ELP10	0.052±0.0025	2.68±0.129	0.434±0.022
TPL-ELP20	0.035±0.0092	2.53±0.609	0.301±0.078
TPL-L6K2	0.013±0.0003	0.513±0.113	0.114±0.002
TPL-EAK16	0.046±0.0028	2.36±0.026	0.389±0.024
TPL-18A	0.045±0.0009	2.18±0.356	0.381±0.007
TPL-GFIL16	0.049±0.0019	2.22±0.014	0.412±0.016

*Symbols " - " represent no detection.
